# Supplementary material for: Serum Metabolomic Analysis of Synchronous Estrus in Yaks Based on UPLC-Q-TOF MS Technology
Source: Animals (Basel). 2024 May 7;14(10):1399. doi: 10.3390/ani14101399 (PMC11117382; doi:10.3390/ani14101399)
Supplement: Supplementary file 1 [file animals-14-01399-s001.zip › animals-2938688-supplementary.pdf]

Table S1. Differential metabolites between GnRH and IO

Metabolite Name	Adduct type	m/z	VIP	FC	*P*.value
(E)-2-Decylpent-2-Enedioic Acid	[M-H]-	269.17639	1.17645	0.563506624	0.00173309
18Alpha-Glycyrrhetinic Acid	[M+ACN+H]+	471.37787	1.3213	2.809077362	0.05902639
3,5-Dihydroxydecanoic Acid	[2M+H]+	227.1257	4.23751	0.73300174	0.001707581
3-Hydroxyproline	[M-H]-	130.0513	1.53887	0.600161382	0.039752088
6-Ethoxy-2-Mercaptobenzothiazole	[M+H]+	212.01976	1.27832	0.621045534	0.022872871
Acacetin	[M-H]-	283.05191	1.00192	0.686931659	0.004529941
Acetylcarnitine	[M]+	204.12233	2.81019	0.776055658	0.162263058
Acetylcholine	[M]+	146.11629	2.31988	0.778526005	0.009948536
Alpha-Hydroxydeoxycholic Acid	[M-H]-	391.30646	1.15619	0.317307109	0.003923365
Auranticin A	[M-H]-	439.22455	1.06097	2.299493938	4.43195E-07
Betaine	[M+H]+	118.08525	6.24555	1.192893972	0.052109384
Bonactin	[M-H]-	399.2775	1.22819	0.315608428	0.022468258
But-3-Enylglucosinolate	[M-H]-	409.98663	1.15401	0.678336622	0.023494538
Butyryl Carnitine	[M+H]+	232.15199	1.32376	0.709205431	0.042908972
Caffeic Acid	[M-H]-	179.03621	1.78844	0.475248194	0.032962228
Capsanthone	[M+H]+	582.42419	1.81824	1.293111182	0.026481774
Chenodeoxycholic Acid	[M+H-2H_2O]+	357.2775	1.48462	0.274921018	0.003322601
Cholesterol	[M+H]+	409.36502	1.7321	0.38453055	6.79652E-05
Cholic Acid	[M+NH_4]+	426.32022	9.83524	0.372028411	0.007941917
Cochlioquinone A	[M-H]-	531.30127	1.15803	0.37697049	0.048958096
Creatinine	[M+H]+	114.06633	8.19833	0.784616691	0.00974309
Dehydrorotenone	[M-H]-	391.22449	1.40835	1.71152947	2.82963E-05
Deoxycholic Acid	[M-H]-	391.28625	4.25864	0.308883693	0.001130748
Deoxysappanone B 7,4'-Dimethyl Ether	[M-H]-	313.19858	1.12738	0.510116959	0.033514089
Dimethyl Sulfoxide	[M+H]+	79.02022	1.21463	1.329978091	0.110013789
Ergosterol Acetate	[M-H]-	465.35959	1.68772	0.399888041	8.03627E-05
Eriodictyol 7,3'-Dimethyl Ether	[2M-H]-	315.08151	2.17636	0.600458748	0.02086942
Eupafolin	[M+H]+	317.05515	1.45889	0.436738577	0.000509594
Gaultherin	[M+H]+	153.06854	1.2227	0.09767635	0.006185389
Glutamate Conjugated Chenodeoxycholic Acid	[M+H]+	544.33789	4.90127	1.421102741	0.020814285
Isocurcumenol	[2M+H]+	235.16641	3.31886	1.569865775	0.042130702
Isorhynchophylline	[M-H]-	383.21198	1.01594	0.498031182	0.036245225
Khasianine	[M+H]+	722.41974	1.4381	0.471008446	0.000660121
Lactic Acid	[M-H]-	89.02409	14.9425	0.50289405	0.026671514
Lichesterylic Acid	[M-H]-	297.24158	0.975147	2.051963549	0.077317431
LPC 16:0	[M+H]+	496.33737	18.4839	1.622153613	0.000167346
LPC 18:1	[M+H]+	522.35468	9.68404	1.179701764	0.024204257
LPE 18:2	[M-H]-	476.27679	3.82294	0.663593136	0.023702205
Microcolin A	[M+H]+	770.56348	1.37865	0.456611805	0.010958611
Microcolin H	[M+ACN+H]+	756.55359	2.03718	0.606774172	0.047148072
Miltirone	[M+Na]+	305.15814	3.20148	1.639737575	0.070670782
Nervonic Acid	[M+H-H2O]+	389.34183	3.67376	0.226589784	5.98775E-05
N-Isovaleroylglycine	[M-H]-	158.08304	2.20756	0.334055419	0.048811359
Octadecanedioic Acid	[M-H]-	313.24176	3.07539	0.750232526	0.016197635
Olmesartan	[M-H]-	445.19513	1.58445	0.296380884	0.036180914
Orlistat	[M+K]+	534.35376	1.06125	1.650967371	0.008795113
Pelletierine Hydrochloride	[M-H]-	176.09242	1.42062	1.464323172	0.076351602

PFCA-Perfluoroalkyl	[M+Hac-H]-	156.99089	2.38158	0.897826541	0.007798167
Phenylacetylglutamine	[M+H]+	265.11752	3.10892	0.271694208	0.107812105
Phenylacetylglycine	[M-H]-	192.06772	4.8509	5.177660809	0.02125045
Phosphatidylcholine Alkenyl 16	[M-H]-	820.5741	1.07825	0.200363987	9.33006E-05
Phosphatidylcholine Lyso 17	[M-H_2O-H]-	538.35059	1.99647	1.541090529	0.000947462
Phosphatidylethanolamine Alkenyl 18	[M-H]-	770.53583	1.1383	0.510488727	0.006591784
Phosphatidylethanolamine Lyso 20	[M-H]-	506.32013	1.10215	1.149230557	0.119252424
Phthalamic Acid	[M-H]-	164.03537	1.04963	2.454596573	0.058021732
Pomiferin	[M-H]-	419.25467	1.33401	2.095870307	0.000437503
Prenyletin	[M-H]-	245.01244	1.0459	0.703027397	0.033979067
Pseudobaptigenin	[M-H]-	281.05371	1.56717	0.752595345	0.010819425
Putative Analogue Of Akanthomycin	[M+NH_4]+	372.2211	1.1775	2.257423475	0.094727954
Ramipril	[M-H]-	415.22592	2.02145	2.17978263	6.64314E-05
Roccellic Acid	[M-H]-	299.22153	1.0969	0.752646265	0.015680933
Secopenitrem D	[M+H]+	570.35138	2.85694	1.65837448	0.046061291
Sn-Glycero-3-Phosphocholine	[M]+	258.10913	0.974209	1.487694801	0.0457497
Sophoridine	[M+H]+	271.16507	1.32192	0.00458436	0.030284269
Sparteine Sulfate	[M+H]+	371.17068	1.47045	0.366600678	0.000426175
Spheroidenone	[M+H]+	582.42798	1.53647	1.955088447	1.29422E-05
Tetracosanoic Acid	[M+H]+	391.35513	1.71432	0.316605267	5.15835E-05
Trigonelline	[M+H]+	138.05437	1.44357	0.241843415	0.022141034
Tryptamine	[M-H]-	159.10263	1.31715	2.025817239	0.089337459
Veratrosine	[M+H]+	572.36774	3.31156	1.832375402	0.005061895

Table S2. Differential metabolites between NE and IO

Metabolite Name	Adduct type	m/z	VIP	FC	*P*.value
2',4',6'-Trihydroxydihydrochalcone	[M-H]-	257.07785	1.09657	1.432448088	0.017652991
2'-Deoxycytidine	[M-H]-	226.08363	1.08111	1.655157951	0.029338332
2-Oxobutyric Acid	[M-H]-	101.02357	1.07544	0.735013101	0.01290469
3-Hydroxyproline	[M-H]-	130.0513	1.67789	1.936905018	0.009613794
3'-Methoxy-4',5,7-Trihydroxyflavonol	[M-H]-	315.0426	2.34783	8.600921268	0.007974719
4-Methylumbelliferyl Sulfate	[M-H]-	254.98244	2.29331	2.011295357	0.001428997
9-Fluorenone	[M-H]-	179.05605	8.89641	0.680377614	0.061539165
9-Trans-Palmitelaidic Acid	[M-H]-	253.2178	1.43718	0.321023698	0.003379803
Acetylcarnitine	[M]+	204.12233	3.00822	1.349982207	0.089993349
Auranticin A	[M-H]-	439.22455	1.16294	0.379822691	1.76623E-10
Azelaic Acid	[M-H]-	187.10043	1.38222	2.109201597	0.032995287
But-3-Enylglucosinolate	[M-H]-	409.98663	1.82169	2.275658699	4.70352E-05
Butyryl Carnitine	[M+H]+	232.15199	1.38128	1.441834348	0.010022187
Caffeic Acid	[M-H]-	179.03621	1.48022	1.797562084	0.04899322
Capsanthone	[M+H]+	582.42419	1.51219	0.80161816	0.013670871
Cholesterol	[M+H]+	409.36502	1.27815	2.003576308	0.006238506
Cholic Acid	[M+NH4]+	426.32022	8.50355	2.357191974	0.055074518
Choline	[M]+	104.10622	2.91115	0.760411858	0.041855765
Citrate	[M-H]-	191.02019	1.70875	1.968959255	0.017188862
Cortisone	[M-H]-	359.19113	1.62946	0.42779568	0.045249186
Dehydrorotenone	[M-H]-	391.22449	1.38674	0.564132967	2.2029E-07
Deoxysappanone B 7,4'-Dimethyl Ether	[M-H]-	313.19858	1.2683	2.220760317	0.010998864
Dl-Beta-Hydroxybutyric Acid	[M-H]-	103.03825	1.44951	8.092587012	0.023875471
Erucamide	[M+H]+	338.34198	1.7099	1.65767815	0.012226516
Eupafolin	[M+H]+	317.05515	1.36068	2.238675615	0.000257118

Feruloyl Lactate	[M-H]-	281.05698	1.17012	4.629722891	0.015480737
FT-Thioether	[M-H]-	187.00737	18.9503	0.750025069	0.028996479
Glutamate Conjugated Chenodeoxycholic Acid	[M+H]+	544.33789	4.68546	0.755268225	0.002695119
Glycocholic Acid	[M-H]-	464.30408	13.5178	1.915459788	0.034859063
Glycolithocholic Acid	[M-H]-	432.31284	1.19674	2.099627574	0.051485056
Glycoursodeoxycholic Acid	[M-H]-	448.3067	9.68418	1.814610018	0.038362659
Hippurate	[M-H]-	178.05156	13.066	34.58032266	0.016710656
Indole + 1O, 1Carboxy, O-Hex	[M-H]-	338.08844	1.62597	0.614235544	0.010259613
Khasianine	[M+H]+	722.41974	1.32373	2.330205622	0.040887492
Lichesterylic Acid	[M-H]-	297.24158	1.08842	0.509771199	0.003220299
LPC 16:0	[M+H]+	496.33737	18.9033	0.584910742	3.34179E-06
LPC 18:1	[M+H]+	522.35468	12.2923	0.759416579	0.012167852
LPE 18:2	[M-H]-	476.27679	3.24919	1.445103353	0.03326406
Microcolin A	[M+H]+	770.56348	1.39294	2.134090713	0.001174046
Microcolin H	[M+ACN+H]+	756.55359	2.46689	1.862910742	0.002545635
N,N-Dimethylaniline	[M+H]+	122.09663	2.68128	1.26435312	0.000767318
N-Cinnamoylglycine	[M-H]-	204.0681	1.23525	2.290133116	0.012617405
Nervonic Acid	[M+H-H_2O]+	389.34183	3.04349	3.254895792	0.000793915
N-Isovaleroylglycine	[M-H]-	158.08304	2.59226	7.042039778	0.004603745
Oleic Acid	[M-H]-	281.24979	2.54948	0.349758918	0.004933513
Olmesartan	[M-H]-	445.19513	1.51035	2.916996341	0.033604461
Palmitic Acid	[M-H]-	255.23274	2.44984	0.66670518	0.011330389
Pannaric Acid	[M-H]-	315.04172	5.57587	1.870756988	0.003941044
PE(16:0/10-Hdohe)	[M-H]-	778.51788	1.11322	1.335164231	0.004587117
PE(18:0/10-Hdohe)	[M-H]-	806.55182	1.3528	1.462743516	7.34828E-05
PE(18:0/11,12-Epete)	[M-H]-	780.51837	1.25379	1.474511312	4.32284E-05
PFCA-Perfluoroalkyl	[M+Hac-H]-	156.99089	2.39412	1.128412324	0.00133699
Phosphatidylcholine Lyso 17	[M-H_2O-H]-	538.35059	2.00212	0.634475621	0.000154307
Phosphatidylethanolamine 18	[M-H]-	792.5343	3.14733	1.273450014	0.007737449
Phosphatidylethanolamine 20	[M-H]-	818.54993	2.43413	1.323475852	6.91948E-05
Phosphatidylethanolamine Alkenyl 18	[M-H]-	770.53583	1.24111	2.666307341	0.000797376
Phosphatidylethanolamine Lyso 17	[M-H]-	466.29688	0.968115	0.736565031	0.003295424
Phosphatidylethanolamine Lyso 20	[M-H]-	506.32013	1.07814	0.861694519	0.082798742
Phosphatidylinositol 18	[M-H]-	885.5473	1.02243	4.427262533	4.24131E-06
Phthalamic Acid	[M-H]-	164.03537	1.24846	0.401463395	0.001697966
Pomiferin	[M-H]-	419.25467	1.10235	0.552845851	2.00096E-05
Pseudobaptigenin	[M-H]-	281.05371	1.01379	1.206731115	0.045044774
Pyroglutamic Acid	[M-H]-	128.0361	3.44397	0.69743175	0.013457297
Ramipril	[M-H]-	415.22592	2.18831	0.394873789	6.74855E-08
Secopenitrem D	[M+H]+	570.35138	3.03624	0.685400372	0.000154821
Sodium Deoxycholate	[M+NH_4]+	393.28513	4.75611	1.238406647	0.006761715
Sparteine Sulfate	[M+H]+	371.17068	1.14058	2.143954917	0.003153934
Spheroidenone	[M+H]+	582.42798	1.23513	0.544839286	0.000151174
Stearic Acid	[M-H]-	283.26547	1.63921	0.774951251	0.054176671
Taurolithocholic Acid	[M-H]-	482.29489	3.42098	1.933386961	0.083489536
Tetracosanoic Acid	[M+H]+	391.35513	1.16672	2.080095486	0.011870374
Thymidine-5'-Monophosphate	[M-H]-	321.04547	0.909258	1.274268503	0.072169103
Trans-Vaccenic Acid	[M-H]-	281.24796	4.07203	0.402237922	0.001643081
Urea	[M+H]+	61.03944	3.46049	0.843386935	0.045511915
Uric Acid	[M+Hac-H]-	167.02126	1.13189	0.487280804	0.00024725

Veratrosine	[M+H]+	572.36774	2.14495	0.706671232	0.008927247
Villol	[M-H]-	441.12433	1.02941	0.507677282	0.00130513
